# Comparison of Reduced-Port Totally Robotic Pancreaticoduodenectomy with Conventional Totally Robotic and Laparoscopic Pancreaticoduodenectomy

**DOI:** 10.3390/jcm14113960

**Published:** 2025-06-04

**Authors:** Boram Lee, Ho-Seong Han, Yoo-Seok Yoon, Jun Suh Lee

**Affiliations:** Department of Surgery, Seoul National University Bundang Hospital, Seoul National University College of Medicine, Seongnam-si 13605, Gyeonggi-do, Republic of Korea; boramsnubhgs@gmail.com (B.L.); yoonys@snubh.org (Y.-S.Y.);

**Keywords:** pancreaticoduodenectomy, laparoscopy, robotic surgical procedures, periampullary neoplasms, minimally invasive surgical procedures

## Abstract

**Background:** Reduced-port totally robotic pancreaticoduodenectomy (rpRPD) has been introduced to address limitations of conventional robotic pancreaticoduodenectomy (cRPD), particularly regarding assistant mobility and visualization. This study aimed to evaluate the clinical feasibility and procedural consistency of rpRPD in comparison with cRPD and laparoscopic pancreaticoduodenectomy (LPD). **Methods**: We conducted a retrospective cohort study of patients who underwent pancreaticoduodenectomy between January 2015 and December 2024. Patients were categorized into rpRPD (n = 40), cRPD (n = 60), and LPD (n = 262) groups. Clinical outcomes and learning curves were compared using regression and cumulative sum (CUSUM) analysis. **Results**: Baseline characteristics were similar across groups. The rpRPD group demonstrated significantly shorter operative time (*p* < 0.001) and lower blood loss (*p* < 0.05) than cRPD, with no significant differences in postoperative complications or hospital stay. The learning curve analysis revealed that rpRPD had lower variance (5839.3 vs. 8919.1) and more stable performance than cRPD despite a slightly longer stabilization point. Lymph node retrieval was comparable across groups, supporting oncological equivalence. **Conclusions**: rpRPD offers comparable perioperative and oncologic outcomes to cRPD and LPD while improving operative efficiency and procedural predictability. It represents a technically feasible and safe option for minimally invasive pancreatic surgery.

## 1. Introduction

Pancreas surgery is inherently difficult, and pancreaticoduodenectomy (PD), also known as the Whipple procedure, is considered one of the most difficult surgeries in this field [1,2]. Traditional open pancreaticoduodenectomy (OPD) has been the standard approach for many years [3]. However, with advancements in surgical technology, minimally invasive surgery (MIS), including laparoscopic pancreaticoduodenectomy (LPD) and robotic pancreaticoduodenectomy (RPD), have gained attention due to their potential benefits in reducing postoperative pain, shortening hospital stay, and accelerating patient recovery [4,5,6]. Moreover, studies have demonstrated that the oncological safety of LPD and RPD is not inferior to that of traditional open surgery [7,8].

RPD sometimes offers distinct advantages over traditional LPD. The robotic system provides enhanced dexterity through articulated instruments, enabling precise and stable dissection and reconstruction in anatomically challenging areas, such as the pancreatic duct and bile duct [9,10]. Despite its distinct advantages, conventional RPD (cRPD) has several limitations that need to be addressed. One of the notable challenges lies in the role of first assistant. Fixed laparoscopic trocars used by assistants often have a limited range of motion due to potential collisions with the robotic arm, limiting the assistant’s ability to make fine adjustments during complex incisions. Additionally, fixating a robotic camera trocar may result in a fixed field of view, potentially impeding the dynamic adjustments needed during complex procedures. To address these issues and optimize the surgical environment, our center introduced a reduced-port totally RPD (rpRPD) utilizing a Glove port.

The rpRPD approach not only alleviates problems caused by limited assistant movement and static camera view but also effectively minimizes surgical scars and postoperative pain by reducing the number of ports. Additionally, the improved range of motion and improved visualization provided by rpRPD allows for more precise and efficient incisions and reconstruction, ultimately contributing to better surgical outcomes and faster patient recovery.

Although several studies have described reduced-port robotic pancreatic surgery [11,12], these have primarily focused on technical feasibility or case series rather than comparative analyses with conventional approaches. In contrast, our study directly compares rpRPD with both cRPD and LPD in terms of clinical outcomes and learning curve profiles. This approach provides new insights into the practical implications of reduced-port techniques within the broader spectrum of minimally invasive pancreatic surgery.

This study aims to evaluate the clinical outcomes, perioperative parameters, and learning curve in patients who underwent rpRPD with patients who received cRPD and LPD. By comparing these three techniques, we aim to reveal the potential advantages of rpRPD, thereby providing valuable insights into the optimization of surgical strategies for periampullary tumors.

## 2. Materials and Methods

This retrospective cohort study was conducted at Seoul National University Bundang Hospital to compare the clinical outcomes, perioperative parameters, and postoperative recovery among patients who underwent rpRPD, cRPD, or LPD. This study included patients aged ≥18 years who underwent PD between January 2015 and December 2024. Patients requiring combined resection of other major organs or with incomplete records were excluded. The study was approved by the Institutional Review Board (IRB) of Seoul National University Bundang Hospital (B-2407-915-101). All procedures followed were in accordance with the ethical standards of the responsible committee on human experimentation and the Helsinki Declaration of 1975, as revised in 2000. As this was a retrospective study and the data were anonymized, the requirement for informed consent was waived by the IRB.

Data were collected retrospectively from medical records, including various aspects such as patient demographics (including age, sex, BMI (Body mass index), preoperative diagnosis, and complications), perioperative data (including operative time, estimated blood loss (EBL), and number of ports used), and postoperative outcomes (including length of hospital stay, postoperative complications, and readmission rates). Oncological outcomes, such as margin status and lymph node yield, were also recorded.

### 2.1. Operative Procedure

**Figure 1:**
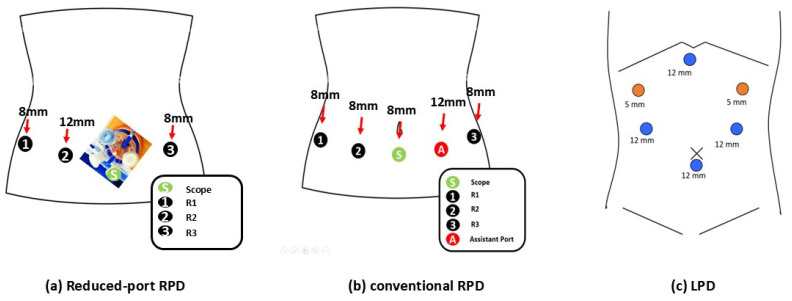
Trocar placement. Schematic representation of trocar placements for (**a**) reduced-port totally robotic pancreaticoduodenectomy (rpRPD), (**b**) conventional totally robotic pancreaticoduodenectomy (cRPD), and (**c**) laparoscopic pancreaticoduodenectomy (LPD).

da Vinci Robotic Surgical System (Intuitive Surgical, Inc) is used to perform RPD. The surgical procedure of rpRPD used in this study was similar to that previously described for performing conventional LPD for periampullary neoplasm [13,14]. Figure 1 shows a schematic diagram showing the overall placement of the rpRPD, cRPD, and LPD. Because the surgical techniques for LPD and cRPD have been extensively documented in previous studies [13,14,15,16], only the rpRPD procedure is described here.

**Figure 2:**
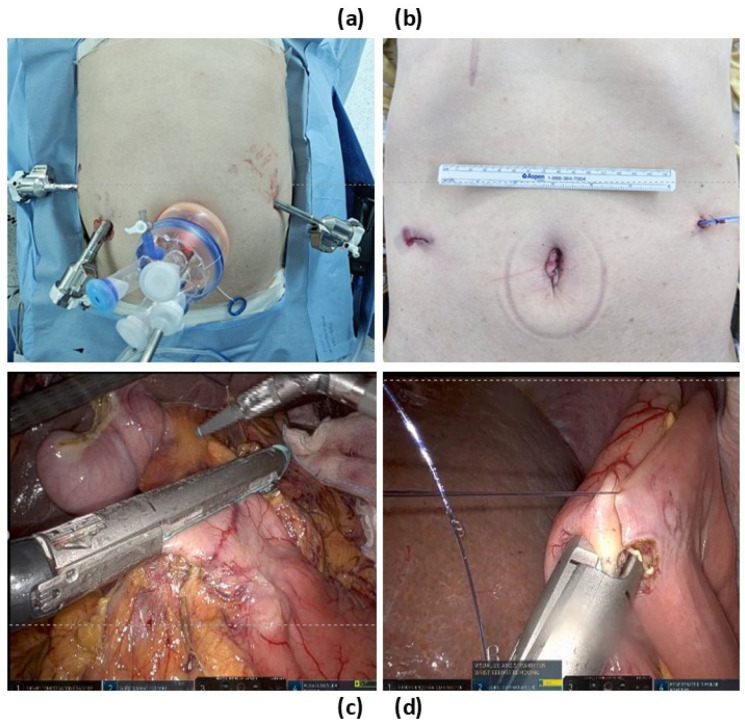
The operative setup and intraoperative views of reduced-port totally robotic pancreaticoduodenectomy (rpRPD). (**a**) The operative setup showing the Glove port placement and robotic arm configuration in rpRPD. (**b**) A postoperative view of the abdomen, demonstrating the reduced number of incisions and the small infra-umbilical incision for the Glove port. (**c**) An intraoperative view showing the use of a robotic stapler through the 12 mm robotic trocar for precise stapling during duodenal transection. (**d**) An intraoperative view of intracorporeal gastrojejunostomy performed using a robotic stapler, demonstrating precise and efficient anastomosis during rpRPD.

The procedure for reduced-port robotic pancreatioduodenectomy (rpRPD) is as follows:

Under general anesthesia, the patient is placed in the lithotomy position with a 30° reverse Trendelenburg tilt with right side up to optimize exposure of the upper abdominal organs. Pneumoperitoneum is established with a CO_2_ pressure of 12 mmHg through an umbilical port. A reduced-port approach is utilized by inserting a Glove port (Inframed, Seoul, Republic of Korea) through a 25 mm vertical infra-umbilical incision. The Glove port accommodates multiple instruments with the following configurations: One 10–15 mm port and three 3–10 mm port. It is primarily use for the camera and first assistant. In addition to the Glove port, the following ports are used: an 8 mm robotic trocar on the right lateral side, a 12 mm robotic trocar on the right medial side to accommodate the SureForm stapler (Figure 2), and an 8 mm robotic port on the left abdomen.

Port configuration and instrumentation is as follows:

The robotic cart is positioned above the patient’s head. The robotic arms are configured as follows:

Arm 1: Tip-up fenestrated grasper in the right 8 mm robotic port.

Arm 2: Fenestrated bipolar forceps or SureForm 60 in the right 12 mm robotic port.

Arm 3: Camera through the 8 mm robotic port in the Glove port.

Arm 4: Vessel Sealer Extend or Maryland bipolar forceps or Monopolar curved scissors in the left 8 mm robotic port.

### 2.2. Statistical Analysis

Descriptive statistics were used to summarize the baseline characteristics of the study population, including age, gender, body mass index (BMI), preoperative diagnosis, and comorbidities. Continuous variables were presented as means and standard deviations or medians and interquartile ranges depending on the data distribution, while categorical variables were expressed as frequencies and percentages. Comparative analyses between the rpRPD, cRPD, and LPD groups were conducted to evaluate differences in perioperative and postoperative outcomes. For categorical variables such as gender distribution, incidence of postoperative complications, and readmission rates, chi-square tests or Fisher’s exact tests were employed. In addition, linear regression was used to analyze early-phase learning curve slopes, while CUSUM analysis was performed to assess variance in the initial phase [15]. Stabilization points were defined as the case number where performance metrics converged within 10% of the mean. Statistical significance was set at *p* < 0.05.

## 3. Results

### 3.1. Baseline Characteristics

The baseline characteristics of the study population were comparable across the rpRPD, cRPD, and LPD groups (Table 1). The majority of patients were male, with proportions of 67.5% in the rpRPD group, 71.6% in the cRPD group, and 68.0% in the LPD group (*p* = 0.238). The mean age was similar among the groups, with 64.5 ± 9.1 years in the rpRPD group, 65.3 ± 11.5 years in the cRPD group, and 64.4 ± 13.1 years in the LPD group (*p* = 0.369). Other factors, including BMI, rates of hypertension and diabetes, ECOG performance status, and tumor characteristics, showed no statistically significant differences between the groups. The tumor location and size were also comparable, with no significant variation in the rates of neoadjuvant chemotherapy (NAT) use (*p* > 0.999).

### 3.2. Postoperative Outcomes

Table 2 shows the operative outcomes between groups. The operative time was significantly shorter in the rpRPD group (420.5 ± 92.2 min) compared to the cRPD group (543.6 ± 117.4 min; *p* < 0.001), while the LPD group had a mean operative time of 444.2 ± 135.7 min. The EBL showed no significant difference in the three-group analysis (*p* = 0.686); however, pairwise comparisons revealed that the EBL in the rpRPD group (326.6 ± 329.2 mL) was significantly lower than in the cRPD group (401.1 ± 383.7 mL). Postoperative outcomes, including the hospital stay, rates of clinically relevant postoperative pancreatic fistula (CR-POPF), and Clavien–Dindo grade complications, showed no statistically significant differences among the groups (*p* > 0.05). Notably, the rpRPD group demonstrated comparable outcomes to cRPD and LPD while requiring less operative time and achieving lower blood loss compared to cRPD.

### 3.3. Learning Curve Analysis

**Figure 3:**
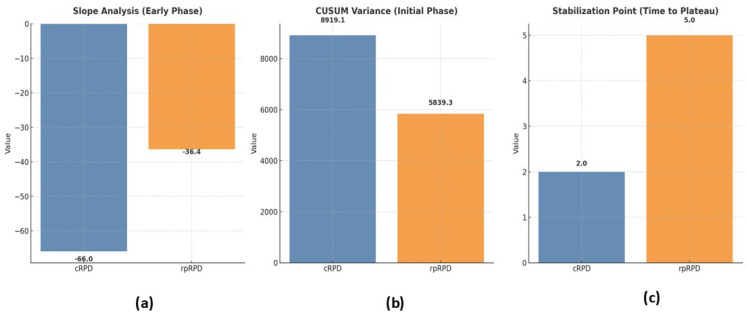
A comparison of learning curves between reduced-port totally robotic pancreaticoduodenectomy (rpRPD) and conventional totally robotic pancreaticoduodenectomy (cRPD). (**a**) The slope analysis (early phase) demonstrates the rate of improvement in operative performance during the early learning phase. rpRPD shows a more gradual slope (−36.4) compared to cRPD (−6.0), indicating steady and consistent learning progress. (**b**) CUSUM variance (initial phase) highlights the variability in performance during the initial learning phase. The variance for rpRPD (5839.3) is significantly lower than for cRPD (8919.1), indicating greater stability and predictability in the early cases of rpRPD. (**c**) The stabilization point (time to plateau) represents the number of cases required to reach consistent operative performance. cRPD stabilizes earlier (2 cases) compared to rpRPD (5 cases), though rpRPD provides smoother procedural improvement over time.

Figure 3 compares the learning curves for rpRPD and cRPD, showing how surgeons adapted when first introduced to each technique and how their skills improved over time with increasing cases. This figure shows the differences in how quickly and consistently surgeons became more proficient in each approach over time. The performance indicators used to calculate these learning curves were the operation time, estimated blood loss (EBL), and hospital stay. The slope analysis for the early phase revealed that rpRPD showed more gradual improvements in performance than cRPD. This demonstrates that the learning process of rpRPD is steady and gradual, allowing surgeons to gradually improve their skills while ensuring the safety and accuracy of the procedure. Conversely, a steeper slope of cRPD suggests that surgeons may be able to improve their skills more quickly initially, but it also means that the skill may be more difficult to learn initially.

The variance in the initial phase, assessed through a CUSUM analysis, was significantly lower for rpRPD. These findings show that rpRPD is safer and more stable in the early stages, as surgeons had less variability in key measures compared to cRPD. Specifically, the higher variance in cRPD (8919.1 vs. 5839.3 for rpRPD) indicates that cRPD had more fluctuations and was less predictable during the initial learning phase. The point of stabilization, a case number indicating the point at which the surgical technique becomes consistent, occurred earlier in cRPD (case 2) than in rpRPD (case 5). While cRPD reached technical stability more quickly, rpRPD required several additional cases to achieve a similar level of performance. Overall, these results suggest that cRPD achieves stabilization more quickly, whereas the rpRPD approach provides superior consistency, greater predictability, and smoother procedural improvement during the learning curve. These properties highlight the potential of rpRPD to improve surgical outcomes and safety, particularly in centers adopting advanced minimally invasive pancreatic surgery.

## 4. Discussion

This study is the first to report on rpRPD, a novel surgical approach developed to improve the difficulties and limitations encountered with conventional multiport RPD. Our results suggest that rpRPD achieves similar perioperative outcomes to cRPD and LPD, while offering distinct advantages in surgical efficiency and procedural consistency. Specifically, rpRPD significantly reduced both the operative time and estimated blood loss (EBL) compared with cRPD, even though the three-group analysis showed no significant difference in the EBL. Additionally, the learning curve analysis results showed that rpRPD had smoother procedural improvements and lower variance in early cases, highlighting its high stability during the introduction period. These results highlight that rpRPD is a viable alternative option to cRPD and LPD, demonstrating the potential to improve surgical outcomes while minimizing the problems associated with RPD.

Previous studies have consistently shown that the operation time for RPD is significantly longer compared to LPD or OPD [16,17,18,19,20]. One inevitable factor contributing to these differences is the time it takes to change robotic equipment, which is inherent in robotic systems [21]. Additionally, when the first assistant lacks sufficient experience, the use of fixed laparoscopic trocars as an assistant may result in significant angulation limitations, which may prolong the surgical time [22]. Furthermore, the rigid robotic scope, once positioned within the fixed trocar, often restricts the flexibility of the field of view. This limitation can make complex reconstructions involving the pancreas or biliary tract more challenging and contribute to longer operation times.

To address these issues, we introduced rpRPD, which integrates a Glove port for improved ergonomics and allows for greater freedom of movement for both the assistant and the camera. This innovative approach aimed to optimize the surgical environment by improving the assistant’s ability to perform fine adjustments and providing dynamic visualization during critical stages of the procedure. Our results confirmed these improvements, as the operative time of rpRPD was significantly shorter than that of cRPD and the preoperative and postoperative outcomes were similar. These study results demonstrate that modifying the port configuration implemented in rpRPD can effectively overcome the limitations of existing RPDs and improve surgical efficiency.

The learning curve analysis demonstrated that rpRPD had lower variability and smoother improvements compared to cRPD, reflecting greater stability during the early adoption phase. One contributing factor to rpRPD’s reduced variability may be its use of a single multiport channel, which minimizes the impact of the first assistant’s skill level. Additionally, the gradual slope of rpRPD suggests that surgeons were able to adapt to the technique steadily without the steep challenges often associated with cRPD. While cRPD reached stabilization earlier, rpRPD offered more consistent progress, emphasizing its potential as a safer and more predictable approach for centers adopting RPD.

This study has several limitations that should be acknowledged. First, the retrospective design and the relatively small sample sizes for the rpRPD and cRPD groups compared to the LPD group may introduce selection bias and limit the statistical power of some comparisons. Second, this study did not evaluate long-term oncological outcomes, leaving open the question of whether rpRPD can be applied more broadly in cancer treatment. Finally, because this study represents an initial evaluation of rpRPD, additional multicenter studies with larger sample sizes and different surgeon experience levels are needed to validate these findings and assess the reproducibility of the technique.

## 5. Conclusions

Despite the limitations of this study, including its retrospective design and lack of long-term oncological outcomes, the results provide compelling evidence that rpRPD can overcome major barriers to RPD and improve procedural consistency. By improving robotic technology and facilitating safer introduction pathways, rpRPD has the potential to improve both surgical outcomes and the overall feasibility of minimally invasive pancreatic surgery. These findings add to the growing body of evidence supporting innovation in robotic surgery and highlight opportunities for further advancement in the field.

## Figures and Tables

**Table 1:** Baseline patient and disease characteristics.

	Patients			
	rpRPD (n = 40)	cRPD (n = 60)	LPD (n = 262)	*p*
Male, N (%)	27 (67.5%)	43 (71.6%)	178 (68.0%)	0.238
Age, mean [SD], years	64.5 ± 9.1	65.3 ± 11.5	64.4 ± 13.1	0.369
BMI, mean [SD], Kg/m^2^	23.4 ± 1.6	24.3 ± 3.0	24.1 ± 4.2	0.595
HTN, N (%)	17 (42.5%)	20 (33.3%)	125 (47.7%)	0.303
DM, N (%)	15 (37.5%)	19 (31.7%)	56 (21.5%)	0.211
ASA, N (%)				0.677
1	11 (27.5%)	15 (25.0%)	54 (20.7%)	
2	25 (62.5%)	40 (66.7%)	172 (65.6%)	
3	4 (2.5%)	5 (8.3%)	36 (13.8%)	
ECOG, N (%)				0.472
0	35 (87.5%)	52 (86.7%)	210 (80.5%)	
1	5 (12.5%)	8 (13.3%)	51 (19.5%)	
Previous obdominal operation hx. N (%)	6 (15%)	17 (28.3%)	88 (33.6%)	0.187
Incidental detection, N (%)	5 (12.5%)	6 (10.0%)	36 (13.7%)	0.701
Weight loss, N (%)	18 (45.0%)	25 (41.7%)	107 (40.8%)	0.881
Jaundice, N (%)	15 (37.5%)	21 (35.0%)	87 (33.2%)	0.931
Biliary Drainage	16 (40.0%)	26 (43.3%)	134 (51.1%)	0.593
Tumor location				0.306
Pancreas	11 (27.5%)	24 (40.0%)	82 (31.3%)	
Comoon bile duct	15 (37.5%)	24 (40.0%)	106 (40.5%)	
AoV	14 (35.0%)	12 (20.2%)	74 (28.2%)	
CEA, mean [SD]	3.8 ± 10.3	4.8 ± 14.1	4.3 ± 21.5	0.883
CA 19-9, mean [SD]	153.5 ± 113.7	369.3 ± 127.1	207.5 ± 755.9	0.638
Tumor size (cm), mean [SD]	2.7 ± 0.9	2.5 ± 1.0	3.04 ± 1.9	0.218
Neoadjuvant CTx, N (%)	0	0	1 (0.4%)	>0.999

rpRPD: Reduced-port robotic pancreaticoduodenectomy; cRPD: conventional robotic pancreaticoduodenectomy; LPD: laparoscopic pancreaticoduodenectomy; SD: standard deviation; BMI: body mass index; HTN: hypertension; DM: diabetes mellitus; ASA: American Society of Anesthesiologists; ECOG: Eastern Cooperative Oncology Group; Hx: history; CEA: carcinoembryonic antigen; CA 19-9: carbohydrate antigen 19-9; CTx: chemotherapy. All variables are presented as the mean and standard deviation or n (%) of patients.

**Table 2:** Operative outcome.

	rpRPD (n = 40)	cRPD (n = 60)	LPD (n = 262)	*p* Value
Op time, mean [SD], min	420.5 ± 92.2	543.6 ± 117.4	444.2 ± 135.7	<0.001
EBL, mean [SD], mL	326.6 ± 329.2	401.1 ± 383.7	362.8 ± 352.7	0.686
Transfusion, N (%)	2 (5.0%)	4 (6.6%)	22 (8.3%)	0.778
Soft pancreas	28 (70.0%)	35 (72.9%)	183 (69.8%)	0.290
P-duct size (mm), mean [SD]	2.62 ± 0.97	2.58 ± 1.89	2.82 ± 1.67	0.888
C-D grade, N (%)				0.244
III	8 (20.0%)	16 (26.6%)	41 (15.6%)	
IV	0	2 (3.3%)	3 (1.0%)	
V	0	0	6 (2.1%)	
CR-POPF, N (%)	8 (20.0%)	10 (20.8%)	47 (17.8%)	0.912
Hopital days, mean [SD], days	12.8 ± 9.3	12.3 ± 11.4	11.6 ± 5.7	0.060
Retrieved Lymph nodes, mean [SD]	17.8 ± 5.2	17.5 ± 6.3	18.1 ± 6.0	0.721

RPD: Robotic pancreaticoduodenectomy; LPD: laparoscopic pancreaticoduodenectomy; SD: standard deviation; Op: operation; EBL: estimated blood loss; P-duct: pancreatic duct; C-D grade: Clavien–Dindo grade; CR-POPF: clinically relevant postoperative pancreatic fistula. All variables are presented as the mean and standard deviation or n (%) of patients.

## Data Availability

The datasets used and analyzed during the current study are available from the corresponding author upon reasonable request.
